# Effects of divided attention on movement-related cortical potential in community-dwelling elderly adults: A preliminary study

**DOI:** 10.1016/j.heliyon.2024.e34126

**Published:** 2024-07-04

**Authors:** Daisuke Hirano, Misaki Wada, Naotoshi Kimura, Daisuke Jinnai, Yoshinobu Goto, Takamichi Taniguchi

**Affiliations:** a Graduate School of Health and Welfare Sciences, International University of Health and Welfare, 4-1-26 Akasaka, Minato, Tokyo, 107-8402, Japan; b Department of Occupational Therapy, School of Health Sciences, International University of Health and Welfare, 2600-1 Kitakanemaru, Otawara, Tochigi, 324-8501, Japan; c Department of Occupational Therapy, School of Health Sciences at Narita, International University of Health and Welfare, 4-3 Kozunomori, Narita, Chiba, 286-8686, Japan; d Department of Physiology, Faculty of Medicine, School of Medicine, International University of Health and Welfare, 4-3 Kozunomori, Narita, Chiba, 286-8686, Japan; e Department of Occupational Therapy, School of Health Sciences at Fukuoka, International University of Health and Welfare, 137-1 Enokizu, Okawa, Fukuoka, 831-8501, Japan

**Keywords:** Movement-related cortical potential, Bereitschaftspotential, Readiness potential, Dual-task interference, Elderly

## Abstract

Dual-tasking is defined as performing two or more tasks concurrently. This study aimed to investigate the effect of divided attention on movement-related cortical potential (MRCP) during dual-task performance in 11 community-dwelling elderly individuals while the load of the secondary task was altered. MRCP was recorded during a single task (ST), simple dual task (S-DT), and complex dual task (C-DT) as no-, low-, and high-load divided attention tasks, respectively. The ST involved self-paced tapping with an extended right index finger. In the S-DT and C-DT, the subjects simultaneously performed the ST and a visual number counting task with different levels of load. The coefficient of variation of movement frequency was significantly more variable in the C-DT than in the ST. The MRCP amplitude from electroencephalography electrode C3, contralateral to the moving hand, was significantly higher in the C-DT than in the ST. Higher attention diversion led to a significant reduction in MRCP amplitude in the participants. These results suggest that attention division in dual-task situations plays an important role in movement preparation and execution. We propose that MRCP can serve as a marker for screening the ability of older individuals to perform dual-tasks.

## Highlights

• We examined the role of attention in neural activity preceding self-paced movements.
• Divided attention levels modulate movement-related cortical potential (MRCP).
• Pre-movement activity is reduced under high-load divided attention in the elderly.
• Divided attention levels influence action preparation-associated brain activity.
• MRCP can serve as a marker for screening risk and safety related to dual-tasks.

## 1 Introduction

In daily life, humans commonly perform multiple tasks simultaneously (multi-tasking), e.g., using a cell phone and walking or driving a car while talking to passengers. However, a previous study reported errors while subjects performed a dual-task of finger tapping and letter counting, although no errors occurred when they performed a single task (either finger tapping or letter counting) [1]. Furthermore, another report showed that when a secondary cognitive task or secondary motor task is added to the Grooved Pegboard Test, which evaluates manual dexterity, the performance of the Grooved Pegboard Test is significantly reduced [2]. These effects can be explained by dual-task interference, which is a reduction in the performance of one or both tasks that are performed concurrently [[3], [4], [5]]. To reduce the risk of accidents, especially in elderly individuals, brain activity could be evaluated before an action is performed in an interventional setting.

One non-invasive technique to assess brain activity related to movement preparation is the analysis of movement-related cortical potential (MRCP), which was selected in this cross-sectional study among various brain function imaging techniques since it can evaluate brain activity before an action is performed. The Bereitschaftspotential or readiness potential, the pre-movement component of MRCP, is a slow cortical potential detected in electroencephalograms that appears a few seconds before a voluntary, self-paced, or imagined movement [[6], [7], [8], [9]]. The primary generators of MRCP are thought to be the bilateral supplementary motor areas (SMAs), bilateral pre-SMAs, bilateral cingulate motor areas, and the contralateral primary motor cortex (M1), with some evidence also suggesting the involvement of the ipsilateral M1 [10]. The magnitude and time course of MRCP varies according to several factors, e.g., preparatory state, precision, complexity, level of intention, learning, movement selection, and praxis movement [7,[11], [12], [13]]. In previous studies focused on the Bereitschaftspotential of self-paced movements using MRCP in dual-task conditions, high-load divided attention in young adults reduced the amplitude of MRCP from electroencephalography electrode C3 of the standard international 10/20 system [14], contralateral to the moving hand [15,16].

The pace of population ageing is increasing, and between 2015 and 2050, the proportion of the world's population aged over 60 years will nearly double from 12 % to 22 % [17]. Aging affects cognitive and motor systems, perturbing the smooth functioning of the cognitive-motor interface [5]. Therefore, it will become increasingly important to ensure the safe and secure lives of the elderly. A previous study implicated attention and working memory processes as critical components of slow movement timing and sources of specific challenges thereof for older adults [18]. In an investigation of age-related differences in the timing control of fast versus slow repetitive movements using a dual-task approach, dual-task costs for both cognitive and timing performances were pronounced at slower tapping tempos, an effect that was exacerbated in older adults [18]. Older adults have increased variability in performance as tapping frequency increases and in the presence of a secondary task. Especially, the performance of older participants is also more variable overall [19]. These studies show that older adults perform worse at dual-tasks compared to younger adults. However, no studies have used MRCP to examine dual-task performance in older adults.

Therefore, the purpose of this study was to investigate the effect of divided attention on MRCP during dual-task performance in community-dwelling elderly individuals while the load of the secondary task was altered to determine if MRCP can serve as a marker for screening the risk and safety related to dual-tasks in interventional settings. Especially, the paradigm used in this study was similar to that employed in a previous study in young adults [16], which strongly recommended that further studies, e.g., a comparison of participants’ age or disease, should be performed to understand better the neural bases of motor preparation and suggested that this paradigm may serve as a marker for screening the capacities of individuals to perform dual tasks. We believe that using the same paradigm will facilitate future comparisons between younger and older adults.

## 2 Materials and methods

### 2.1 participants

Eleven right-handed elderly individuals living in the community (two females and nine males; mean age, 74 ± 4 years; range, 68–81 years) volunteered to participate in the study. The participants were recruited from the Silver Human Resource Center, which supports the employment of Japan's older workers. Our study was approved by the Institutional Ethics Committee of the International University of Health and Welfare, Japan (approval no. 22-Io-29-2). Written informed consent was obtained from all participants prior to their participation in the study.

The participants were included if they were able to perform the basic and instrumental activities of daily living (BADLs and IADLs, respectively) and were employed. We excluded individuals with cognitive decline, dementia, mild cognitive impairment, mental disorder, or visual impairments that may affect their performance of the study tasks. We examined the BADLs and IADLs and performed neuropsychological testing according to a previous study (Table 1 [20]). The BADLs and IADLs were assessed using the Barthel Index (BI) [21], Lawton's IADL Rating Scale [22], Japan Science and Technology Agency Index of Competence [23], and Tokyo Metropolitan Institute of Gerontology Index of Competence [24]. The neuropsychological tests included the Japanese version of the Montreal Test of Cognitive Abilities [25,26], Verbal Fluidity Test [27], and Trail Making Test Parts A and B [28].

**Table 1:** Demographic data of the participants.

No.	Sex	Age (y)	Education (y)	BI	Lawton	JST-IC	TMIG-IC	MoCA-J	VFT	TMT-A	TMT-B
1	F	75	12	100	8	9	12	29	4	43	104
2	M	68	12	100	5	15	13	29	15	49	79
3	M	73	16	100	5	13	13	26	19	27	105
4	M	70	12	100	5	13	12	27	8	43	66
5	M	68	16	100	5	16	13	26	11	51	80
6	F	76	12	100	8	15	13	24	9	41	53
7	M	72	12	100	5	16	13	26	12	26	92
8	M	81	12	100	5	14	13	22	8	60	144
9	M	75	9	100	5	12	13	20	9	63	88
10	M	79	12	100	5	15	13	27	12	49	78
11	M	75	12	100	5	15	13	30	13	35	88
AV		74	12	100	*	14	13	26	11	44	89
SD		4	2	0	0	2	0	3	4	11	23

AV: average; BI: Barthel Index [21]; F: Female; JST-IC: Japan Science and Technology Agency, Index of Competence [24]; Lawton: Lawton's IADL Rating Scale [22]; M: Male; MoCA-J: Japanese version of Montreal Cognitive Assessment [25,26]; SD: standard deviation; TMIG-IC: Tokyo Metropolitan Institute of Gerontology, Index of Competence [23]; TMT-A: Trail making test A [28]; TMT-B: Trail making test B [29]; VFT: Verbal fluency task [27]; y: years. *The maximum score for a female and a male is 8 and 5 points, respectively [22].

### 2.2 Tasks

**Fig. 1:**
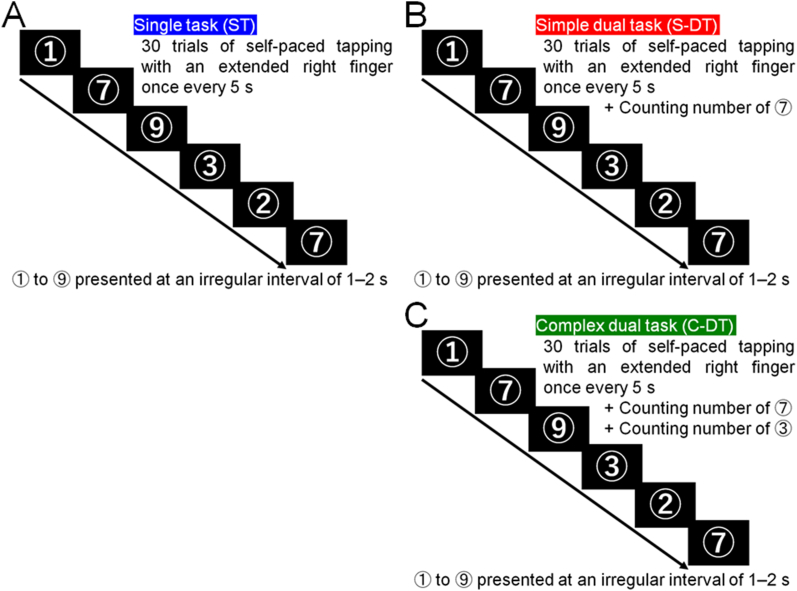
Schematics showing the structure of a typical trial in the single task (ST; A), simple dual task (S-DT; B), and complex dual task (C-DT; C).

The participants performed three tasks: a single task (ST) and a dual task (DT) consisting of a simple DT (S-DT) and a complex DT (C-DT). The three testing conditions were performed in the order of the ST, S-DT, and C-DT. The ST was a motor task as a no-load divided attention task that involved three blocks with 30 trials of self-paced tapping with an extended right index finger once every 5 s (Fig. 1A). The S-DT was a low-load divided attention task that consisted of three blocks with 30 trials of self-paced tapping with an extended right index finger once every 5 s combined with a simple number counting task, which involved counting the number of times a target number appeared among nine numbers, with the numbers presented at an irregular interval of 1–2 s (Fig. 1B). The C-DT was a high-load divided attention task that consisted of three blocks with 30 trials of self-paced tapping with an extended right index finger once every 5 s combined with a complex number counting task, which involved counting the number of times two target numbers appeared among nine numbers (Fig. 1C). After each block, the subjectss were asked to answer orally the number of times each target number appeared. The X-type Continuous Performance Test (CPT) in the Clinical Assessment for Attention (Japan Society for Higher Brain Dysfunction, Tokyo, Japan) was used in the visual number counting task. Before the first session, the subjects performed the motor task until self-paced tapping with an extended right index finger once every 5 s could be performed correctly ∼5 times and they were briefed about the method to execute the tasks.

The paradigm in this study was used similar to that employed in previous studies in young adults to investigate the effect of divided attention on MRCP during dual-task performance [15,16,29,30]. Especially, in younger healthy adults, the MRCP amplitude from C3 electrode of the international 10/20 system [14] is significantly higher in the S-DT than in the ST, whereas it is similar between the ST and C-DT. The S-DT causes significantly more changes in the MRCP magnitude than ST [16].

### 2.3 Recording

Each participant was seated in a comfortable chair located 50–60 cm away from a digital computer monitor and their right and left forearms were placed on a desk. A surface electromyography electrode (Polymate Mini AP108; Miyuki Giken, Tokyo, Japan) was placed on the extensor digitorum muscle of the index finger to determine action onset. An active electroencephalogram electrode system was used two monopolar electroencephalogram channels (Polymate Mini AP108; Miyuki Giken, Tokyo, Japan) from C3 and C4, according to the international 10/20 system [14] and the reference electrodes were placed on both earlobes [16]. MRCP amplitude in finger movements is highest at the electroencephalography electrode C1 or C2 of the international 10/10 system [31,32]. We adopted the international 10/20 system [14] because it has gained importance as a standard head surface-based relative positioning method in various transcranial brain mapping methods [33]. The C3 and C4 electrodes of the international 10/20 system [14] are the left and right lateral electrodes closest to the C1 and C2 electrodes of the international 10/20 system, respectively [14]. Since MRCP reflects the process of motor preparation [[6], [7], [8], [9], [10], [11], [12], [13]], it is important to investigate MRCP separately in the SMA, pre-SMA, and M1. However, in this study, we also minimized the number of electroencephalogram electrodes to measure MRCP easily in individuals with various diseases and disabilities in different communities in the future. Therefore, the activity of the SMA, pre-SMA, and M1 could not be separated and electroencephalography electrodes C3 and C4, which are closest to the M1, were selected.

### 2.4 Analysis

The accuracy of the number of target numbers identified correctly under the S-DT and the C-DT conditions was assessed in the visual number counting task. The frequency of finger tapping in the ST, S-DT, and C-DT was quantified by considering the coefficient of variation (CV = standard deviation/average) between action onset in each task condition. The MRCP signal was digitized at 500 Hz, with an amplifier band-pass from 0.05 to 10 Hz [16,29] and a 50-Hz notch filter [34] to remove power supply noise from lighting, etc. Further, to reduce high-frequency noise, a Savitzky-Golay filter (polynomial order: 5; window width: 0.3) was used for additional waveform smoothing on the time-averaged MRCPs. The baseline was calculated from 3 to 2.5 s before action onset. Data were segmented into epochs from 3 s prior to action onset to 1 s after it. Trials with electrooculography artifacts were excluded by using a threshold of 1 mV. The amplitude and latency of peak negativity were considered as the initial features.

### 2.5 Statistical analysis

The results are shown in the text as the mean ± standard deviation. To compare the accuracy of the number of target numbers identified correctly under the S-DT and C-DT conditions, the Wilcoxon signed-rank test was used. For the comparison of the CV of finger tapping in the ST, S-DT, and C-DT, Friedman's test was used. Bonferroni's *post-hoc* test was used in multiple pairwise comparisons. Friedman's test was used to analyze the amplitude and latency of peak negativity to determine the effect of the divided attention tasks on MRCP. All statistical analyses were performed with SPSS28 software (IBM Corporation, Armonk, NY, USA) with the significance level set at *p* < 0.05.

## 3 Results

### 3.1 Task performance

**Fig. 2:**
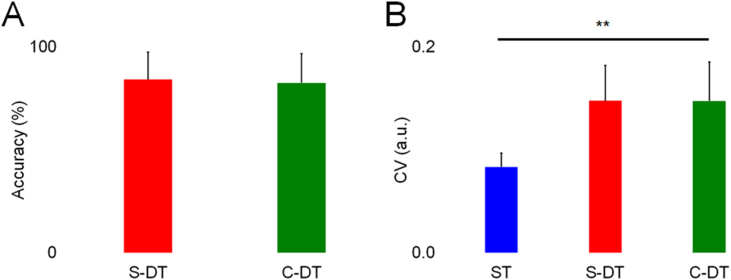
Accuracy of the visual number counting tasks (A) and coefficient of variation (CV) of movement frequency (B). ST: single task; S-DT: simple dual task; C-DT: complex dual task. **p < 0.001.

The accuracy of the visual number counting tasks and the CV of movement frequency in the ST, S-DT, and C-DT are displayed in Fig. 2A and B. The accuracy was not affected in the S-DT and C-DT (S-DT: 84 ± 14 %, C-DT: 83 ± 15 %, *p* = 0.889). Friedman's test revealed a significant main effect for the CV of movement frequency (*χ*^*2*^ = 9.333, *p* = 0.009). The CV was significantly more variable in the C-DT (0.13 ± 0.04 arbitrary units [a.u.]) than in the ST (0.08 ± 0.01 a.u., *p* = 0.012). There was no difference in the CV of movement frequency between the S-DT (0.13 ± 0.03 a.u.) and ST (0.08 ± 0.01 a.u., *p* = 0.063) and between the S-DT (0.13 ± 0.03 a.u.) and C-DT (0.13 ± 0.04 a.u., *p* = 1.000).

### 3.2 Pre-movement brain activity

**Fig. 3:**
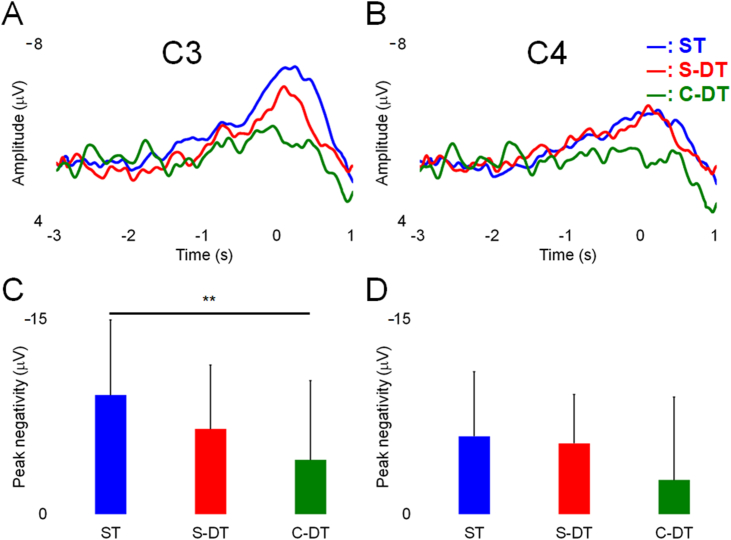
Grand average of movement-related cortical potential (MRCP) from all participants obtained from electroencephalography electrode C3 (A) and C4 (B) of the standard international 10/20 system [14] and amplitude of peak negativity from C3 (C) and C4 (D). ST: single task; S-DT: simple dual task; C-DT: complex dual task. **p < 0.001.

The average MRCP from encephalography electrodes C3 and C4 in the ST, S-DT, and C-DT across all participants is shown in Fig. 3A and B. Friedman's test revealed a significant main effect for the amplitude of peak negativity on C3 (*χ*^*2*^ = 10.333, *p* = 0.006). The amplitude on C3 was higher in the ST (−9 ± 6 μV) than in the C-DT (−4 ± 7 μV, *p* = 0.004, Fig. 3C). No significant differences were found in the latency of peak negativity on C3. No significant variations in the amplitude and latency of peak negativity on C4 were found in both tasks (Fig. 3D).

## 4 Discussion

The purpose of this study was to examine the effects of dual-task complexity on the pre-movement brain activity associated with readiness for action in elderly people living in the community, specifically examining the influence of divided attention load on MRCP. To our knowledge, this is the first investigation of the effects of dual-task complexity in elderly individuals. Overall, we found that pre-movement brain activity was significantly reduced under conditions of high-load divided attention (i.e., C-DT) compared to no-load divided attention (i.e., ST).

In this study, the CV of movement frequency was significantly more variable in the C-DT than in the ST. These behavioral data can be explained by dual-task interference, which is a reduction in the performance of one or both tasks that are performed concurrently [[3], [4], [5]]. In the present study, attention was divided using a visual number counting task while the participants performed a simple self-timed finger movement as the main task. The performance of this task was understood to be influenced by the secondary (visual number counting) task via attention diversion. In particular, our results suggested that the CV of motor frequency was significantly more variable in the C-DT than in the ST.

The amplitude of peak negativity on encephalography electrode C3 was significantly higher in the C-DT than in the ST, whereas it was similar between the S-DT and ST and between the C-DT and S-DT. Our results were similar to those of previous MRCP studies using a dual task in young adults [15,16]. Pre-movement neural activity in the MRCP is attenuated when cognitive resources are not readily available for movement preparation, and early stage premovement activity reflects the engagement of specific cognitive processes that overlap with cognitive control and working memory [15]. We consider that the MRCP amplitude was higher in the ST than in the C-DT because the cognitive resources of the may not be readily available for action preparation in the C-DT. This results suggest that the MRCP amplitude during motor preparation can be used to detect the divided attention changes of participants, specifically at different levels of task load [16].

Our results in elderly adults showed no significant differences in behavior and MRCP amplitudes and latencies between the ST and S-DT. Conversely, a previous study using a similar task in young adults found a significant difference in MRCP amplitude between the ST and S-DT [16]. We hypothesize that this difference reflects the fact that the amount of attentional deviation allocated to the ST and S-DT is similar in elderly adults, whereas it is different in young adults. Ageing is accompanied by functional dysregulation of motor cortex excitability during sensorimotor processing, with this deficit becoming progressively evident with greater task complexity [35]. This result suggests that elderly adults require the same level of attention in the ST as in the S-DT.

Our MRCP results are similar to those of studies examining the characteristics of P300 wave amplitude, which is an event-related potential component elicited during decision making. In previous dual-task studies, P300 amplitude decreases when performing a dual task since it is more difficult than executing a single task [36,37]. P300 amplitude is also larger when more attention is needed and decreases when a task is more complex [[38], [39], [40]]. P300 amplitude is related to the attention system and working memory [41] and may be an indicator of neural power or cognitive resources, which increases with maturation [42]. P300 latency represents information processing time [41] and may be an indicator of neural speed or brain efficiency [42]. Therefore, the amplitude of MRCP contralateral to the moving hand, similar to the amplitude of P300, is thought to reflect distributed attentional resources.

We suggest that the functions of the prefrontal cortex (PFC), which is related to time perception, including the dorsolateral prefrontal cortex (DLPFC), which is related to working memory, and the SMA, which is related to intentional performance, and these networks influenced the amplitude of MRCP in this study according to the findings of the following studies. During preparation for a voluntary action, activity in the SMA consistently precedes activity in the M1 prior to the initiation of a movement, which has been replicated in studies using functional magnetic resonance imaging [43], positron emission tomography [44], and magnetoencephalography [45]. The self-paced tapping with an extended right index finger once every 5 s used in this study largely involves the SMA and DLPFC [46]. Studies on motor timing show that premovement activity in the SMA is affected when maintaining movement rhythm in the absence of external cues [47]. Motor timing deficits have been reported in patients with SMA lesions [48]. The SMA controls movement initiation in difficult sequences that have to fit into a precise timing schedule [49]. The SMA activation is more increased during interval timing [50,51]. The SMA is also more active during intentional performance than during automatic performance [52,53] or a distractor task [54]. Moreover, the SMA is also involved in selecting the correct moment to initiate an action [51,55] and in orienting attention to points in time [56,57]. Therefore, our results suggest that our motor task reflects the function of the SMA.

PFC lesions reduce neuronal input into the SMA, and this deficit in the preparatory motor network may cause the reduction of MRCPs observed in patients with traumatic frontal brain injury [58]. In timing tasks, the DLPFC is responsible for maintaining a representation of a given duration and attending to presented stimuli in working memory [50,[59], [60], [61], [62], [63]]. Greater activation is observed in the DLPFC than in other regions during timing tasks in healthy individuals [[64], [65], [66]]. Additionally, the PFC is implicated in time perception in pharmacological [67] or lesion studies [68]. Decreases in DLPFC activation are observed in patients with schizophrenia in the CPT [69]. In working memory tasks, an increase in the GABAergic DLPFC neurons firing rate is detected during a delay [70]; GABA increases the synchronization of pyramidal cell neurons, increasing DLPFC activation and facilitating task performance in healthy individuals [70]. DLPFC activation is also seen when subjects pay attention while performing a pre-learned sequence of movements compared to when they perform the sequence automatically [52]. Several previous studies have emphasized the importance of the DLPFC in attention to action [71].

Lowered MRCP amplitudes are suggested to reflect a decrease in M1 activity to some extent [72]. Attention can alter neuronal activity not only in higher motor areas but also within the M1 [73]. The M1 is differentially activated when attention is directed toward an action compared to when it is not [74]. Although a meta-analysis related to M1 activity during a dual task also showed a reduction of M1 inhibition during the task, no common consensus has been reached regarding cortical activity during a dual task with an added cognitive component [75]. For example, a significant reduction in the functional magnetic resonance imaging signal for both motor movement and distractor tasks compared with that for only a motor movement task in the M1 was reported [76]. The M1 is also activated when attention is not required for an action when participants perform a visual task while constantly moving their index finger [77]. An additional task may result in a reduced ability to activate the M1 inhibitory networks [78]. Motor evoked potential during different difficulty dual tasks shows an increase in M1 inhibition during an easier dual task [79]. The motor evoked potential facilitated in M1 during finger movement with tactile discrimination is substantially reduced when a cognitive task is added [80]. Especially, older adults seemed to adopt a “cognitive-first” prioritization strategy during a dual task, and deficits in dual task performance may be related to the modulation of M1 inhibitory mechanisms [81]. A significant relationship between poorer dual task motor performance in older adults and a reduction in inhibitory control within the M1 are observed [82]. The control of motor performance is dependent on the activation of not only excitatory neurons but also inhibitory neurons within the M1 [83,84], so interference caused by a concurrent task may result in the under activation of inhibitory neurons within the M1 [82].

Only a few studies of MRCP have been performed in elderly individuals. Regarding the amplitude of MRCP, it has often been reported that there is no difference between elderly and young people [[85], [86], [87], [88]]. Conversely, movement processes show age-dependent decreases with higher MRCP amplitudes, likely reflecting the less efficient recruitment of the neuronal resources required for the execution of a task [89]. Latency is reportedly both longer [87,88] or not longer [85,86] in elderly subjects compared to young people. It has been hypothesized that the elderly require a longer time to prepare for voluntary movements than young individuals, which may be the reason for the prolonged latency of MRCP [88]. The present study did not directly compare elderly and young individuals. However, our results demonstrate that, regardless of age, MRCP amplitudes occurring contralaterally to the moving hand can be used during action preparation to assess participants' divided attention changes at different levels of task complexity. In the future, in addition to comparisons with young people, we should compare elderly subjects and individuals with mild cognitive impairment and attention disorders.

This study has some limitations. First, since the participants were recruited through the Silver Human Resource Center, which supports the employment of older workers in Japan, they were elderly people living in the community who were active and had similar high levels in the BADLs and IADLs. This study also had a small sample size. The generalizability of our findings requires further investigation with larger sample sizes and a wider range of community-dwelling elderly individuals. Secondly, the order of the tasks was the same across subjects, so there may be order effects; therefore, it will be important to verify our findings using a task paradigm that avoids order effects. Thirdly, only the electrical activity of C3 and C4 in the international 10/20 system [14] was used. Therefore, the activity of the SMA, pre-SMA, and M1 could not be separated. As a next step, measurements with a higher spatial resolution and detailed analysis are required. Finally, since this study was cross-sectional in design, the data acquired were from elderly people at a certain point in time. Therefore, we intend to investigate longitudinal progress including real-world settings, e.g., the relationship between car driving and accidents.

Several models have tried to explain dual tasks and their effects in humans. Despite decades of investigation, our understanding of the neurobiological basis of dual-task performance is still limited [[3], [4], [5],^90^]. Cognitive-motor interference has been assessed using various human brain mapping techniques to measure related brain activity [3]. However, there is no consensus as to which theory best predicts the effects of dual tasks [3,91,92]. Investigations of the behavioral and neuronal correlates of dual-task performance in animals have suggested how dual tasks are processed in the brain [90]. Studies have also shown that dual-task training preserves or improves physical and cognitive function in older adults and people with neurological disorders [93,94]. Improvement of dual-task performance in individuals with neurologic disorders may improve gait, balance, and cognition [95]. Our results will have important implications as MRCP can serve as a marker for screening risk and safety related to dual- or multi-tasks in real-life settings, since the modulation of cortical plasticity is affected by attention [[96], [97], [98]].

## 5 Conclusions

We explored the effect of internal attention diversion on movement execution characteristics in elderly adults. Our results show that the CV of movement frequency was significantly more variable under attention diversion. High-load attention diversion led to a significant reduction in MRCP amplitude in elderly people. High-load divided attention had a pronounced effect on the movement preparation phase in the elderly. The next step is to investigate the effects of attention diversion associated with dual tasks when performing the BADLs and IADLs.

## Funding

This work was supported by the International University of Health and Welfare.

## Data availability statement

The data, analytical methods, and study materials may be made available to other researchers. Interested researchers should contact the corresponding author. These data are not available to the public to protect the privacy of the study participants. The authors do not have permission to share data.

## Ethical statement

Our study was approved by the Institutional Ethics Committee of the International University of Health and Welfare, Japan (approval no. 22-Io-29-2). Written informed consent was obtained from all participants prior to their participation in the study. The participants could withdraw consent at any point prior to the publication of the results. We confirm that we have read the Journal's position on issues involved in ethical publication and affirm that this work is consistent with those guidelines.

## CRediT authorship contribution statement

**Daisuke Hirano:** Writing – review & editing, Writing – original draft, Methodology, Investigation, Conceptualization. **Misaki Wada:** Writing – review & editing, Validation, Methodology, Investigation, Formal analysis. **Naotoshi Kimura:** Writing – review & editing, Validation, Methodology, Investigation, Formal analysis. **Daisuke Jinnai:** Writing – review & editing, Validation, Methodology, Investigation, Formal analysis. **Yoshinobu Goto:** Writing – review & editing, Supervision, Methodology, Conceptualization. **Takamichi Taniguchi:** Writing – review & editing, Supervision, Methodology, Conceptualization.

## Declaration of competing interest

The authors declare that they have no known competing financial interests or personal relationships that could have appeared to influence the work reported in this paper.
